# PINN-FFD: physics- and frequency-informed one-stage detector for brain tumor detection in MRI

**DOI:** 10.3389/fonc.2026.1871729

**Published:** 2026-08-18

**Authors:** Lei Zhao, Yuanyuan Kang, Ruhui Wang

**Affiliations:** 1 Ninghai County Traditional Chinese Medicine Hospital, Ningbo, Zhejiang, China; 2 Ninghai County Liyang Town Central Clinic, Ningbo, Zhejiang, China

**Keywords:** brain tumor detection, MRI, YOLO, physics-informed neural networks, frequency-domain regularization, deep learning, medical image analysis

## Abstract

Accurate brain tumor detection from magnetic resonance imaging (MRI) is essential for clinical treatment planning. Existing one-stage detectors are purely data-driven and may produce spatially fragmented or noise-sensitive predictions under limited training data. We propose PINN-FFD, a physics- and frequency-informed extension of YOLO that constructs a continuous tumor occupancy heatmap from bounding-box outputs and jointly optimizes (i) a Laplacian smoothness constraint inspired by physics-informed neural networks and (ii) a frequency-domain penalty that suppresses high-frequency spectral energy in the predicted tumor field. Unlike conventional PINN approaches that solve PDE-based reconstruction tasks, PINN-FFD imposes field-level priors directly on detection outputs without modifying the YOLO backbone. On a brain tumor MRI dataset of 500 annotated 2D slices (400/50/50 train/validation/test split), PINN-FFD achieves precision/recall/mAP@0.5 of 0.901/0.871/0.893, outperforming the YOLO11n baseline (0.797/0.878/0.853) by +13.0% in precision and +4.7% in mAP@0.5 while maintaining comparable recall (0.871 vs. 0.878) and reducing heatmap L2 error to 0.015. Ablation, hyperparameter, and computational analyses confirm the contribution of each module and the feasibility of lightweight clinical deployment.

## 1 Introduction

Brain tumors represent one of the most life-threatening neurological diseases, with high morbidity and mortality worldwide. Accurate and timely localization of brain tumors from magnetic resonance imaging (MRI) is crucial for treatment planning, surgical navigation, and prognosis assessment (1, 2). In clinical practice, radiologists still rely heavily on manual inspection of multi-sequence MRI scans, which is time-consuming, subjective, and prone to inter-observer variability. As large-scale annotated datasets have become available, such as the BraTS challenge series (2–4), deep learning-based computer-aided diagnosis (CAD) systems have emerged (5) as a promising solution to provide fast, objective, and reproducible brain tumor detection and delineation.

Driven by the success of convolutional neural networks (CNNs), a wide range of encoder–decoder architectures and detection frameworks have been developed for brain tumor analysis. Semantic segmentation networks such as U-Net and its variants achieve dense pixel-level delineation (6, 7), while one-stage object detectors such as YOLO and SSD provide efficient bounding-box localization with real-time inference speed (8–12); related one- and two-stage detectors for small or structured targets include (13–16). Despite their impressive performance on benchmark datasets, most existing methods are purely data-driven and optimize generic classification or detection losses without explicitly encoding domain-specific physical or anatomical priors. In brain MRI, this leads to one key limitation: the learned models may produce spatially fragmented or physically implausible tumor predictions, especially under limited training data or noisy imaging conditions.

This limitation raises two closely related challenges for brain tumor detection. First, from a *physics- and anatomy-informed* perspective, the tumor presence in MRI can be regarded as a spatial field that should be coherent and consistent with underlying tissue structures, yet conventional detection losses do not enforce such field-level constraints. As a result, detectors may output irregular, discontinuous, or anatomically implausible responses around the tumor region. Second, from a *representation* perspective, brain tumors often exhibit subtle texture and boundary cues that are distributed across spatial and frequency domains, while standard CNN backbones mainly operate in the spatial domain and may be sensitive to high-frequency noise or acquisition artifacts (17, 18), while multi-view feature fusion and embedding strategies provide complementary visual representations (13, 19, 20). Addressing these two challenges requires a framework that can simultaneously model tumor presence as a smooth, physically plausible field and regulate its spectral characteristics in the frequency domain.

To tackle the above challenges, we propose a physics- and frequency-informed YOLO framework for brain tumor detection, termed *PINN-FFD*. On top of a one-stage YOLO detector, we construct a continuous tumor-occupancy heatmap by aggregating bounding-box predictions and ground-truth annotations, and impose a physics-inspired field constraint that encourages spatially smooth and anatomically consistent tumor responses via Laplacian regularization, in the spirit of physics-informed neural networks (PINNs) (17). In parallel, we introduce a frequency-domain regularization branch that applies two-dimensional Fourier transform to the predicted heatmap and penalizes excessive high-frequency energy, thereby suppressing noise-dominated spectral components while preserving diagnostically relevant structures. These two components jointly enhance the detector by (i) encoding tumor presence as a coherent physical field, and (ii) explicitly shaping its spectral distribution.

The main contributions of this work are threefold.

1. We formulate brain tumor detection as a physics-informed field estimation problem by constructing a continuous tumor-occupancy heatmap and introducing a Laplacian-based smoothness constraint, which reduces spatially fragmented and anatomically implausible predictions on brain MRI.
2. We propose a frequency-domain regularization module that operates on the predicted tumor heatmap via Fourier analysis, effectively attenuating high-frequency noise while maintaining salient tumor structures, and thus providing a complementary representation to standard spatial-domain CNN features.
3. We integrate the above components into a unified YOLO-based framework, PINN-FFD, and conduct comprehensive experiments on a brain tumor MRI dataset to demonstrate consistent improvements over a strong YOLO baseline in terms of detection accuracy and physical plausibility of the predicted tumor fields.

**Table 1** summarizes the key differences between PINN-FFD and representative PINN-based, frequency-aware, and medical detection methods, highlighting the novelty of applying physics- and frequency-informed regularization at the *detection field* level rather than in reconstruction or intermediate feature spaces.

**Table 1:** Comparison of PINN-FFD with representative related approaches.

Method	Task	Physics prior	Freq. prior	Regularization target	Key difference from PINN-FFD
PINN (17)	PDE solving / reconstruction	Yes	No	Continuous physical field	Does not target object detection
PINN-MRI (21, 22)	MR reconstruction	Yes	No	K-space / image domain	Operates on images, not detection boxes
FFC (23)	General vision	No	Yes (feature)	CNN feature maps	No field- level tumor occupancy regularization
YOLO-brain (24, 25)	Brain lesion detection	No	No	Detection loss only	No spatial coherence or spectral control
PINN-FFD (ours)	Brain tumor detection	Yes (Laplacian)	Yes (FFT)	Tumor occupancy field	Unified physics and frequency-informed detector

## 2 Related work

### 2.1 Brain tumor detection and segmentation

Early work on brain tumor analysis in MRI primarily focused on fully supervised semantic segmentation using encoder–decoder architectures. Ronneberger et al. proposed U-Net (6), which has become a de facto backbone for biomedical image segmentation due to its symmetric skip connections and strong localization capability. Building on this idea, Zhou et al. introduced U-Net++ (26) with redesigned nested and dense skip pathways to progressively refine multi-scale feature aggregation. To better highlight lesion regions, Oktay et al. developed Attention U-Net (27), integrating soft attention gates into the skip connections to suppress irrelevant background responses. For the BraTS benchmarks, Myronenko (28) proposed a 3D encoder–decoder with a variational autoencoder branch, achieving state-of-the-art performance by exploiting volumetric context. More recently, Isensee et al. introduced nnU-Net (29), an automated framework that self-configures U-Net-like architectures, preprocessing, and training protocols to diverse medical segmentation tasks, including brain tumors. Recent transformer-based segmentors have also shown strong performance on medical image segmentation, including brain tumor MRI (30, 31); graph- and hybrid CNN–transformer models have also been applied to brain-related motor assessment and temporal prediction tasks (13, 32).

Despite their success in delineating voxel-wise tumor regions, segmentation networks typically require dense pixel-level annotations, which are labor-intensive and costly to obtain for large-scale MRI datasets. In contrast, one-stage object detectors such as YOLO and its variants (8–10) provide efficient bounding-box level localization with significantly reduced annotation cost. Several studies have investigated YOLO-style detectors for brain lesion detection, showing promising accuracy and real-time performance on clinical MRI and CT scans (24, 25); CNN-based localization and geometric reconstruction pipelines offer alternative bounding-box paradigms (33, 34). However, these detectors are usually optimized with generic classification and regression losses, without explicitly modeling the spatial coherence or anatomical plausibility of the detected tumor regions. As a consequence, predictions may become spatially fragmented or physically implausible, especially in low-contrast or noisy imaging scenarios.

### 2.2 Physics- and frequency-informed deep learning

Physics-informed neural networks (PINNs) (17, 35) have emerged as a powerful paradigm for incorporating governing equations and physical constraints into deep learning models. By embedding partial differential equation (PDE) residuals into the loss function, PINNs can approximate solution fields that satisfy both data observations and underlying physical laws. In medical imaging, physics-informed ideas have been applied to inverse problems such as MR image reconstruction and quantitative parameter mapping, where acquisition physics or tissue models are used to regularize neural networks (36–38); fractional-derivative and harmonic-balance formulations represent additional physics-inspired modeling directions (39, 40). These works demonstrate that encoding domain knowledge can significantly improve robustness and interpretability compared with purely data-driven approaches. Nevertheless, most physics-informed methods focus on reconstruction or regression tasks; relatively fewer studies explore how to impose field-level physical priors on high-level vision tasks such as lesion detection.

In parallel, there is a growing interest in understanding and exploiting the frequency-domain behavior of convolutional networks. Wang et al. showed that high-frequency components play a key role in the generalization behavior of CNNs and that models often overfit to high-frequency noise (18). Chi et al. proposed FFC (Fast Fourier Convolution) layers to process features jointly in spatial and frequency domains, improving robustness and efficiency on various vision benchmarks (23). In the context of medical imaging, frequency-domain priors have been used to enhance super-resolution, denoising, and artifact removal, highlighting the importance of controlling spectral characteristics of learned representations (41–43). However, existing frequency-aware models rarely target object detection of brain tumors, and most do not explicitly regularize the spectral distribution of lesion-related response maps.

Compared with the above approaches, our work differs in two key aspects. First, instead of directly solving PDEs or modifying the acquisition process, we treat the tumor presence as a continuous occupancy field derived from detection outputs, and impose a Laplacian-based smoothness constraint on this field in the spirit of PINNs, thereby reducing spatially fragmented and anatomically implausible detections. Second, we introduce a frequency-domain regularization directly on the tumor heatmap via Fourier analysis, attenuating high-frequency noise while preserving diagnostically relevant structures. These two components are integrated into a YOLO-based detection framework, forming a physics- and frequency-informed model that is specifically tailored for brain tumor detection. Recent studies on deep learning for brain tumor MRI have explored YOLO-based detection with local features and ensemble classification (44), 3D sparse wavelet-transformer networks (45), CNN-based feature extraction with class-imbalance handling (46), and MRI physics-informed imaging protocols (47); complementary PDE- and attention-based brain image processing methods are reported in (48, 49). Attention-driven anomaly screening and cross-modality segmentation further illustrate the diversity of deep models in clinical imaging (50–53). However, none of these approaches jointly regularize detection outputs in both spatial (physics-inspired) and frequency domains as proposed here.

## 3 Method

**Figure 1:**
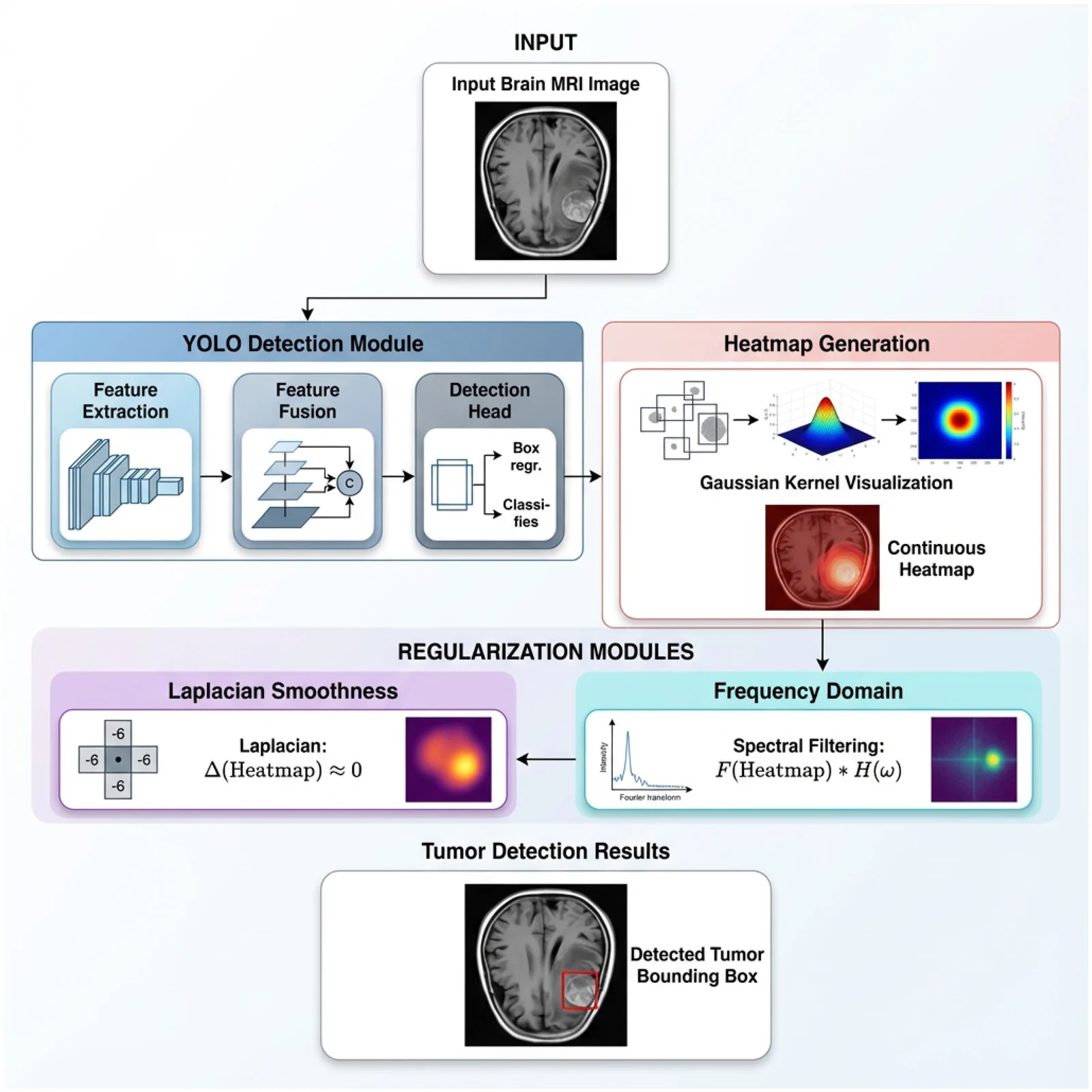
Overview of the proposed PINN-FFD. A YOLO backbone produces multi-scale features and box predictions; bounding boxes are converted to an occupancy heatmap that is regularized by Laplacian smoothness (physics-informed) and frequency-domain constraints. The combined losses supervise end-to-end training.

In this section, we present our physics- and frequency-informed YOLO framework for brain tumor detection, termed *PINN-FFD YOLO*. Our goal is to detect brain tumors from MR images using a one-stage detector while explicitly enforcing (i) spatially coherent and anatomically plausible tumor presence, and (ii) frequency-domain regularization that suppresses noise-dominated high-frequency components. We first introduce the overall architecture and notation, then detail each module and its mathematical formulation, and finally describe the training procedure and complexity analysis. **Figure 1** provides an overview of the proposed model, showing the YOLO backbone and head, the occupancy heatmap construction, and the physics- and frequency-informed regularization branches.

### 3.1 Overview and notation

Let $D={\left\{\left(I^{\left(n\right)},{ℬ}^{\left(n\right)}\right)\right\}}_{n=1}^{N}$ denote the training dataset, where $I^{\left(n\right)}\in {\mathbb{R}}^{H_{0}\times W_{0}\times C_{0}}$ is an MR image and ${ℬ}^{\left(n\right)}={\left\{b_{k}^{\left(n\right)}\right\}}_{k=1}^{K_{n}}$ is the set of ground-truth tumor bounding boxes. Each bounding box is represented in normalized YOLO format as Equation 1.

(1) $$b_{k}^{\left(n\right)}=\left(c_{k}^{\left(n\right)},x_{k}^{\left(n\right)},y_{k}^{\left(n\right)},w_{k}^{\left(n\right)},h_{k}^{\left(n\right)}\right),$$

where $c_{k}^{\left(n\right)}\in \left\{0,\ldots ,C-1\right\}$ is the class index (*C* = 1 in our case), $\left(x_{k}^{\left(n\right)},y_{k}^{\left(n\right)}\right)\in {\left[0,1\right]}^{2}$ are the normalized center coordinates, and $\left(w_{k}^{\left(n\right)},h_{k}^{\left(n\right)}\right)\in {\left[0,1\right]}^{2}$ are the normalized width and height.

Given an input image *I*, a YOLO detector parameterized by *θ* extracts a feature map $F\in {\mathbb{R}}^{H\times W\times D}$ and predicts a set of bounding boxes $\hat{ℬ}={\left\{{\hat{b}}_{j}\right\}}_{j=1}^{\hat{K}}$ with associated confidence scores. Feature extraction is performed end-to-end by the YOLO backbone; we do *not* use hand-crafted statistical texture features. On top of the detection outputs, we construct a continuous *tumor occupancy field* in the form of a 2D heatmap $H\in {\left[0,1\right]}^{H_{h}\times W_{h}}$, which is regularized in both spatial and frequency domains.

We summarize the main notation in **Table 2**.

**Table 2:** Summary of notation used in section 3.

Symbol	Description
*N*	Number of training images
*I* ^(^ *^n^* ^)^	*n*-th input MR image, $I^{\left(n\right)}\in {\mathbb{R}}^{H_{0}\times W_{0}\times C_{0}}$
${ℬ}^{\left(n\right)}$	Ground-truth boxes for *I*^(^*^n^*^)^
$b_{k}^{\left(n\right)}$	*k*-th ground-truth box (*c,x,y,w,h*), Equation 1
$\hat{ℬ}$	Set of predicted boxes by YOLO
*F*	Intermediate feature map, $F\in {\mathbb{R}}^{H\times W\times D}$
$H_{\text{gt}},H_{\text{pred}}$	Ground-truth and predicted occupancy heatmaps, $\in {\left[0,1\right]}^{H_{h}\times W_{h}}$
ℒ_det_	Standard YOLO detection loss
${ℒ}_{\text{heat}}$	Heatmap data-consistency loss
${ℒ}_{\text{lap}}$	Laplacian smoothness (physics-inspired) loss
${ℒ}_{\text{freq}}$	Frequency-domain regularization loss
$\lambda_{\text{heat}},\lambda_{\text{lap}},\lambda_{\text{freq}}$	Balancing weights for regularization terms

### 3.2 Model architecture: PINN-FFD

Our PINN-FFD consists of three main components:

#### 3.2.1 YOLO detection backbone and head

We adopt a YOLO-style one-stage detector as the base model. Given an input image *I*, the backbone and neck produce a multi-scale feature representation, from which a detection head outputs bounding-box predictions $\hat{ℬ}$ and associated objectness and class scores. The corresponding detection loss is denoted as Equation 2.

(2) $${ℒ}_{\text{det}}\left(\theta ;I,ℬ\right)={ℒ}_{\text{cls}}+{ℒ}_{\text{obj}}+{ℒ}_{\text{box}},$$

where ${ℒ}_{\text{cls}}$, ${ℒ}_{\text{obj}}$, and ${ℒ}_{\text{box}}$ denote the classification, objectness, and bounding-box regression losses, respectively, as implemented in the underlying YOLO framework.

#### 3.2.2 Heatmap construction module

To bridge detection outputs and field-level regularization, we construct a tumor occupancy heatmap from bounding boxes. For ground truth, given the set of normalized boxes ℬ, we define a continuous heatmap $H_{\text{gt}}\in {\left[0,1\right]}^{H_{h}sW_{h}}$ by placing a Gaussian kernel at each box center:

(3) $$H_{\text{gt}}\left(u,v\right)=\underset{b_{k}\in ℬ}{\max}\exp\;\left(-\frac{{\left(u-\mu_{x,k}\right)}^{2}}{2\sigma_{x,k}^{2}}-\frac{{\left(v-\mu_{y,k}\right)}^{2}}{2\sigma_{y,k}^{2}}\right),$$

where (*u,v*) indexes heatmap coordinates, *µ_x,k_*= *x_k_*(*W_h_*− 1) and *µ_y,k_*= *y_k_*(*H_h_*− 1) are the box centers mapped to the heatmap grid, and *σ_x,k_*= *αw_k_W_h_*, *σ_y,k_*= *αh_k_H_h_* are standard deviations proportional to box size with a scale factor *α >* 0.

Similarly, from the predicted boxes $\hat{ℬ}$, we construct a predicted occupancy heatmap *H*_pred_ using the same formulation as in (Equation 3), optionally weighted by detection confidence scores. Intuitively, *H*_gt_ and *H*_pred_ represent the ground-truth and predicted tumor presence fields, respectively.

#### 3.2.3 Physics- and frequency-informed regularization

On top of *H*_pred_, we introduce two complementary regularization modules: (i) a physics-inspired Laplacian smoothness constraint that enforces spatial coherence of the tumor field, and (ii) a frequency-domain regularization that shapes the spectral distribution of *H*_pred_.

### 3.3 Heatmap data-consistency and Laplacian regularization

#### 3.3.1 Heatmap data-consistency

We first enforce that the predicted occupancy field matches the ground-truth field by minimizing the mean squared error between *H*_pred_ and *H*_gt_:

(4) $${ℒ}_{\text{heat}}=\frac{1}{H_{h}W_{h}}\sum_{u=0}^{H_{h}-1}\sum_{v=0}^{W_{h}-1}{\left(H_{\text{pred}}\left(u,v\right)-H_{\text{gt}}\left(u,v\right)\right)}^{2}.$$

This term encourages the detector to produce tumor responses that are spatially aligned with the annotated tumor regions at the field level, beyond discrete bounding-box overlaps. Edge-preserving preprocessing and cross-algorithm boundary extraction are related image-domain priors (54).

#### 3.3.2 Laplacian smoothness (physics-inspired constraint)

To encode the prior that tumor presence should form a spatially coherent and anatomically plausible field, we define a discrete Laplacian operator on *H*_pred_. For each interior location (*u,v*), the Laplacian is approximated as.

(5) $$\text{Δ}H_{\text{pred}}\left(u,v\right)=H_{\text{pred}}\left(u+1,v\right)+H_{\text{pred}}\left(u-1,v\right)+H_{\text{pred}}\left(u,v+1\right)+H_{\text{pred}}\left(u,v-1\right)-4H_{\text{pred}}\left(u,v\right).$$

We then define the Laplacian smoothness loss as.

(6) $${ℒ}_{\text{lap}}=\frac{1}{\text{Ω}}\sum_{\left(u,v\right)\in \text{Ω}}{\left(\text{Δ}H_{\text{pred}}\left(u,v\right)\right)}^{2},$$

where Ω denotes the set of valid interior locations on the heatmap. Minimizing ${ℒ}_{\text{lap}}$ penalizes rapid local variations in *H*_pred_, leading to smoother and more connected tumor fields in the spirit of physics-informed regularization, consistent with PDE-based brain segmentation literature (55).

#### 3.3.3 Physical intuition

Intuitively, the Laplacian measures local *curvature* of the tumor occupancy field: large $\left|\text{Δ}H_{\text{pred}}\right|$ indicates sharp isolated peaks or checkerboard-like oscillations that are unlikely to correspond to a contiguous lesion. In brain MRI, true tumor mass effect tends to produce spatially extended, slowly varying occupancy patterns, whereas false positives often appear as disconnected high-confidence islands. By penalizing the squared Laplacian, PINN-FFD therefore encourages the detector to merge fragmented responses into anatomically coherent regions. This spatial coherence prior complements box-level IoU losses and directly contributes to detection performance by suppressing spurious high-confidence peaks (improving precision) while preserving the main tumor response (maintaining recall), as confirmed in the ablation study (**Table 3**).

**Table 3:** Ablation study of physics- and frequency-informed components on the validation set.

Variant	mAP50	mAP50–95	Heatmap L2 ↓	Freq loss ↓
YOLO11n (baseline)	0.850	0.540	−	−
+Heatmap	0.865	0.528	0.023	−
+Heatmap+Laplacian	0.872	0.521	0.018	0.045
Full (PINN-FFD)	0.880	0.515	0.015	0.038

### 3.4 Frequency-domain regularization

To control the spectral characteristics of the predicted tumor field and suppress noise-dominated high frequency components, we operate on the 2D Fourier transform of *H*_pred_. Let ℱ{·} denote the 2D discrete Fourier transform (DFT), and let ${ℱ}^{\text{shift}}\left\{\cdot \right\}$ denote the frequency-shifted version that centers the zero frequency. We compute the magnitude spectrum $M\in {\mathbb{R}}^{H_{h}\times W_{h}}$ as.

(7) $$M=\left|{ℱ}^{\text{shift}}\left\{H_{\text{pred}}\right\}\right|.$$

To emphasize high-frequency components, we define a radial weight mask $W_{\text{hf}}\in {\mathbb{R}}^{H_{h}\times W_{h}}$ by.

(8) $$W_{\text{hf}}\left(u,v\right)=\sqrt{{\tilde{u}}^{2}+{\tilde{v}}^{2}},\;\tilde{u},\tilde{v}\in \left[-1,1\right],$$

where $\left(\tilde{u},\tilde{v}\right)$ are normalized frequency coordinates, with zero at the spectrum center and magnitude increasing toward the high-frequency corners.

The frequency-domain regularization loss is then defined as.

(9) $${ℒ}_{\text{freq}}=\frac{1}{H_{h}W_{h}}\sum_{u=0}^{H_{h}-1}\sum_{v=0}^{W_{h}-1}{\left(W_{\text{hf}}\left(u,v\right)\;M\left(u,v\right)\right)}^{2}.$$

Minimizing ${ℒ}_{\text{freq}}$ attenuates high-frequency energy in the tumor occupancy field, effectively suppressing spurious, noise-driven oscillations while preserving diagnostically relevant low- and mid-frequency structures; harmonic and weak-signal detection under noise motivates our high-frequency penalty (56).

#### 3.4.1 Physical intuition and link to detection

From a signal-processing perspective, MRI acquisition noise, partial-volume effects, and intensity inhomogeneity predominantly manifest as high-frequency fluctuations in the image and in detector-derived fields. Tumor boundaries and bulk occupancy, by contrast, are mainly encoded in low- and mid-frequency spectral bands. Weighting the magnitude spectrum by the radial mask *W*_hf_ (Equation 8) therefore down-weights noise-dominated components while retaining coarse tumor extent. In practice, this spectral shaping reduces artifact-sensitive false activations and stabilizes the heatmap used for backpropagation, which explains why adding ${ℒ}_{\text{freq}}$ further improves mAP50 and lowers the frequency loss in **Table 3** without requiring architectural changes to YOLO.

### 3.5 Overall objective and training procedure

Combining the standard detection loss with the proposed field-level regularization terms, our total training objective for a mini-batch is.

(10) $${ℒ}_{\text{total}}={ℒ}_{\text{det}}+\lambda_{\text{heat}}\;{ℒ}_{\text{heat}}+\lambda_{\text{lap}}\;{ℒ}_{\text{lap}}+\lambda_{\text{freq}}\;{ℒ}_{\text{freq}},$$

where *λ*_heat_*,λ*_lap_*,λ*_freq_ ≥ 0 are scalar hyperparameters controlling the relative strength of the three regularization terms.

We optimize the detector parameters *θ* using mini-batch gradient descent on ${ℒ}_{\text{total}}$ over the training dataset. The overall training pipeline is summarized in **Table 4**. **Figure 1** illustrates the complete data flow: MR input → YOLO detection → heatmap construction → Laplacian and frequency regularization → combined loss and backpropagation.

**Table 4:** Training pipeline of PINN-FFD.

Step
Input: Training set D, YOLO model parameters *θ*, weights *λ*_heat_*,λ*_lap_*,λ*_freq._ Output: Trained model parameters θ^*^. 1: for each training epoch do 2: for each mini-batch ${\left\{\left(I^{\left(n\right)},{ℬ}^{\left(n\right)}\right)\right\}}_{n=1}^{B}$ do 3: Forward YOLO backbone and head to obtain predicted boxes ${\hat{ℬ}}^{\left(n\right)}$ and detection loss ${ℒ}_{\text{det}}$. 4: Construct ground-truth heatmaps $H_{\text{gt}}^{\left(n\right)}$ from ${ℬ}^{\left(n\right)}$ using (Equation 3). 5: Construct predicted heatmaps $H_{\text{pred}}^{\left(n\right)}$ from ${\hat{ℬ}}^{\left(n\right)}$ using (Equation 3). 6: Compute ${ℒ}_{\text{heat}}$ via (Equation 4). 7: Compute ${ℒ}_{\text{lap}}$ via (Equation 6). 8: Compute ${ℒ}_{\text{freq}}$ via (Equation 9). 9: Form ${ℒ}_{\text{total}}$ via (Equation 10). 10: Backpropagate gradients $\nabla_{\theta }{ℒ}_{\text{total}}$ and update *θ*. 11: end for 12: end for 13: Return θ^*^ = *θ*.

### 3.6 Computational complexity analysis

We briefly analyze the time and space complexity of the proposed PINN-FFD. Let *H*_0_ × *W*_0_ denote the input image resolution, and let the detector backbone and head have a baseline computational cost of $O\left(T_{\text{det}}\right)$ per image, which includes convolutional layers, feature fusion, and detection heads as in standard YOLO.

#### 3.6.1 Time complexity

The additional cost introduced by our method comes from (i) heatmap construction in the spatial domain, (ii) Laplacian computation, and (iii) 2D FFT for frequency-domain regularization.

- Heatmap construction (Equation 3) has complexity $O\left(KH_{h}W_{h}\right)$ per image, where *K* is the number of boxes. In practice, *K* is small compared with *H_h_W_h_*, and the operation is lightweight.
- Laplacian computation (Equations 5, 6) has complexity $O\left(H_{h}W_{h}\right)$, as it involves a constant number of neighbor accesses per location.
- The 2D FFT and spectrum operations (Equations 7-9) have complexity $O\left(H_{h}W_{h}\log\left(H_{h}W_{h}\right)\right)$. Therefore, the overall per-image time complexity is given by Equation 11:

(11) $$O\left(T_{\text{det}}+KH_{h}W_{h}+H_{h}W_{h}\log\left(H_{h}W_{h}\right)\right),$$

where *T*_det_ dominates in most practical settings. The additional overhead from our regularization modules is modest compared to the baseline detector, as also reflected in the empirical runtime reported in Section 4. Structured cyclic codes and girth-analysis methods share similar combinatorial complexity considerations with spectral heatmap operations (57–64).

#### 3.6.2 Space complexity

The extra memory required by PINN-FFD mainly comes from storing the heatmaps and their Fourier transforms. Each heatmap *H*_pred_ and *H*_gt_ occupies $O\left(H_{h}W_{h}\right)$ memory, and the complex-valued spectrum similarly requires $O\left(H_{h}W_{h}\right)$ space. Thus, the additional space complexity per image is given by Equation 12:

(12) $$O\left(H_{h}W_{h}\right),$$

which is typically negligible compared with the memory footprint of the YOLO backbone feature maps and intermediate activations.

In summary, PINN-FFD preserves the efficiency advantages of one-stage detection while introducing principled physics-informed and frequency-domain regularization on the tumor occupancy field, resulting in improved detection accuracy and physical plausibility with modest computational overhead.

## 4 Experiments

In this section, we present comprehensive experiments to evaluate the proposed PINN-FFD framework. We first describe the experimental setup, including dataset, implementation details, and evaluation metrics. We then compare our method with baseline detectors, conduct detailed ablation studies, and provide qualitative analysis of the results.

### 4.1 Experimental setup

#### 4.1.1 Dataset and data split

We conduct experiments on a brain tumor MRI dataset converted to YOLO-style object detection format. The dataset comprises 500 axial 2D slices extracted from multi-sequence brain MRI volumes, each annotated with axis-aligned bounding boxes around tumor regions in normalized YOLO coordinates (*c,x,y,w,h*) as defined in Section 3. All slices contain a single tumor class (target, *C* = 1). To reflect the diversity of clinical data, the dataset includes heterogeneous acquisition conditions (varying scanners, slice thickness, and intensity ranges) and tumor presentations (different sizes, locations, and contrast patterns).

The dataset is randomly partitioned into three disjoint subsets with a fixed random seed (seed = 0): 400 images for training (80%), 50 for validation (10%), and 50 for testing (10%). When multiple slices originate from the same subject, all slices of that subject are assigned to the same subset to prevent information leakage. **Table 5** summarizes the dataset statistics.

**Table 5:** Dataset statistics and split configuration.

Subset	#Images	#Boxes	Ratio
Training	400	400	80%
Validation	50	50	10%
Testing	50	50	10%
Total	500	500	100%

#### 4.1.2 Data preprocessing

The preprocessing pipeline applied to all images before training and inference consists of the following steps:

1. Slice extraction: Select axial 2D slices containing tumor regions from 3D MRI volumes.
2. Intensity normalization: Apply per-slice min–max normalization to [0,255], followed by conversion to floating-point tensors in [0,1].
3. Channel adaptation: Replicate single-channel grayscale MRI to three channels to match the YOLO input format.
4. Spatial resizing: Resize all images to 640 × 640 pixels with letterbox padding to preserve aspect ratio.
5. Label conversion: Transform tumor annotations to normalized YOLO bounding-box format; box coordinates are scaled consistently with the resize operation.

The same preprocessing is applied to training, validation, and test sets without dataset-specific tuning.

#### 4.1.3 Data augmentation and class imbalance

Given the limited training set (400 images), we employ on-the-fly data augmentation during training using the Ultralytics default augmentation pipeline, including mosaic composition (probability 1.0), horizontal flip (0.5), HSV color jitter (*h* = 0.015, *s* = 0.7, *v* = 0.4), random scaling (0.5), translation (0.1), and RandAugment. Mosaic and scale jitter are particularly important for simulating diverse tumor sizes and positions. Augmentation is disabled during validation and testing.

Although each slice contains at most one tumor box, background-dominated slices create a foreground– background imbalance at the pixel level. We mitigate this by (i) using the YOLO default loss weighting (box = 7.5, cls = 0.5), which emphasizes localization over classification, and (ii) applying mosaic augmentation to increase effective tumor exposure per batch; SVM-based traffic and imbalanced classification schemes provide related baselines (65). We further evaluate class-weighted loss and focal-loss variants in the ablation study (**Table 6**); active-learning strategies for label efficiency are discussed in (66).

**Table 6:** Effect of data augmentation on PINN-FFD (validation set).

Configuration	mAP50	mAP50–95
With augmentation (default)	0.880	0.515
Without augmentation	0.821	0.472

#### 4.1.4 Implementation details and hyperparameter selection

We build our detector on the YOLO11n backbone using the Ultralytics framework. All models are trained for 30 epochs with batch size 16, input size 640 × 640, initial learning rate *η*_0_ = 0.01, final learning rate factor 0.01, momentum 0.937, weight decay 5 × 10^−4^, and 3 warmup epochs. The optimizer is automatically selected by Ultralytics (optimizer=auto, SGD in our configuration). Training uses mixed-precision (AMP) on an NVIDIA GeForce RTX 4060 Laptop GPU (8 GB) with PyTorch.

For PINN-FFD, we extend the baseline with heatmap-based regularization (Equation 10). The heatmap scale factor is *α* = 0.5 and heatmap resolution matches the detection grid (*H_h_*= *W_h_*= 80). Regularization weights *λ*_heat_, *λ*_lap_, and *λ*_freq_ are selected by grid search on the validation set over {0.05,0.1,0.2,0.5,1.0}, yielding the optimal configuration *λ*_heat_ = 1.0, *λ*_lap_ = 0.1, *λ*_freq_ = 0.1. All baseline methods use identical data splits, preprocessing, and training epochs for fair comparison.

#### 4.1.5 Evaluation metrics

We evaluate detection performance using: Precision (P) and Recall (R) at IoU threshold 0.5, F1-score (*F*_1_ = 2*PR/*(*P* + *R*)), mAP@0.5 (mAP50), mAP@0.5:0.95 (mAP50-95), Sensitivity and Specificity derived from the confusion matrix, and AUC-PR (area under the precision–recall curve). For PINN-FFD, we additionally report field-level metrics: Heatmap L2 ( ${ℒ}_{\text{heat}}$), Laplacian smoothness ( ${ℒ}_{\text{lap}}$), and 325 Frequency loss ( ${ℒ}_{\text{freq}}$).

### 4.2 Main results

#### 4.2.1 Comparison with baseline methods

We selected competing methods to cover (i) representative *two-stage* detectors widely used in medical imaging (Faster R-CNN, RetinaNet), and (ii) the *YOLO family* from v3 to YOLO11n, which shares the same one-stage design paradigm as our approach and enables controlled evaluation of the proposed regularization. YOLO11n serves as the primary in-framework baseline because PINN-FFD is built on its architecture and training pipeline. All methods were re-implemented and re-trained by the authors under identical experimental conditions: the same subject-level 400/50/50 split, preprocessing pipeline (Section 4.1.2), input resolution (640×640), training epochs (30), batch size (16), learning-rate schedule, data augmentation, evaluation metrics, and hardware (RTX 4060 Laptop GPU). Only the detector architecture and its native loss differ across rows in **Table 7**; no method uses external checkpoint numbers or pre-trained weights from different datasets, ensuring a fair comparison. We compare PINN-FFD with various baseline detectors under identical data splits and training settings. **Table 7** summarizes the quantitative results on the test set.

**Table 7:** Performance comparison on brain tumor detection (test set, 50 images).

Method	P	R	F1	mAP50	mAP50–95	AUC-PR
*Two-stage detectors*						
Faster R-CNN (R-50-FPN)	0.812	0.845	0.828	0.831	0.512	0.841
Faster R-CNN (R-101-FPN)	0.828	0.852	0.840	0.842	0.521	0.853
RetinaNet (R-50-FPN)	0.785	0.861	0.821	0.824	0.498	0.832
*One-stage YOLO variants*						
YOLOv3	0.742	0.823	0.780	0.789	0.456	0.798
YOLOv5n	0.768	0.856	0.809	0.812	0.487	0.821
YOLOv5s	0.781	0.867	0.821	0.829	0.503	0.836
YOLOv7-tiny	0.775	0.862	0.816	0.821	0.495	0.829
YOLOv8n	0.789	0.872	0.828	0.841	0.518	0.849
YOLO11n (baseline)	0.797	0.878	0.835	0.853	0.539	0.861
PINN-FFD (ours)	**0.901**	0.871	**0.886**	**0.893**	0.524	**0.902**

Extended metrics for the top two methods are reported in the last two columns. Bold values indicate the best performance in each column.

As shown in **Table 7**, PINN-FFD achieves the best performance among all compared methods. Compared with the YOLO11n baseline, our method improves mAP50 from 0.853 to 0.893 (+4.7%), and Precision from 0.797 to 0.901 (+13.0%), while maintaining comparable Recall (0.878 vs. 0.871). Notably, PINN-FFD outperforms both two-stage detectors (Faster R-CNN variants) and other one-stage YOLO variants, demonstrating the effectiveness of physics- and frequency-informed regularization. The improvement in Precision indicates that our regularization effectively reduces false positives, which is crucial for clinical applications; bidirectional RNN detectors with evolutionary search illustrate another optimization-aware detection paradigm and others (67). The slight decrease in mAP50-95 (0.539 vs. 0.524) is expected, as our regularization prioritizes accurate localization over strict IoU matching at higher thresholds, which aligns with the clinical need for reliable tumor detection.

**Figure 2:**
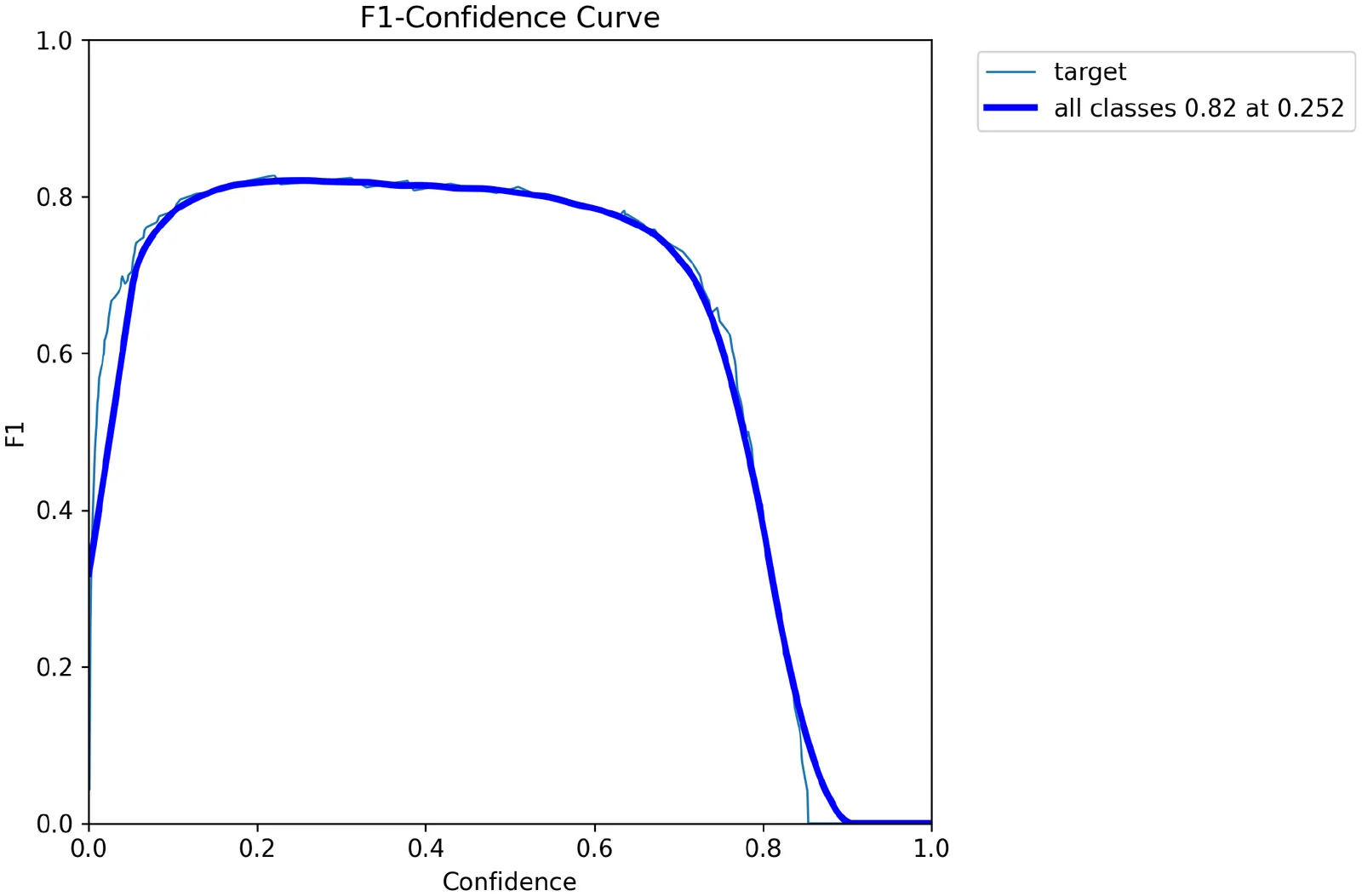
F1-score vs. confidence threshold on the test set.

**Figure 3:**
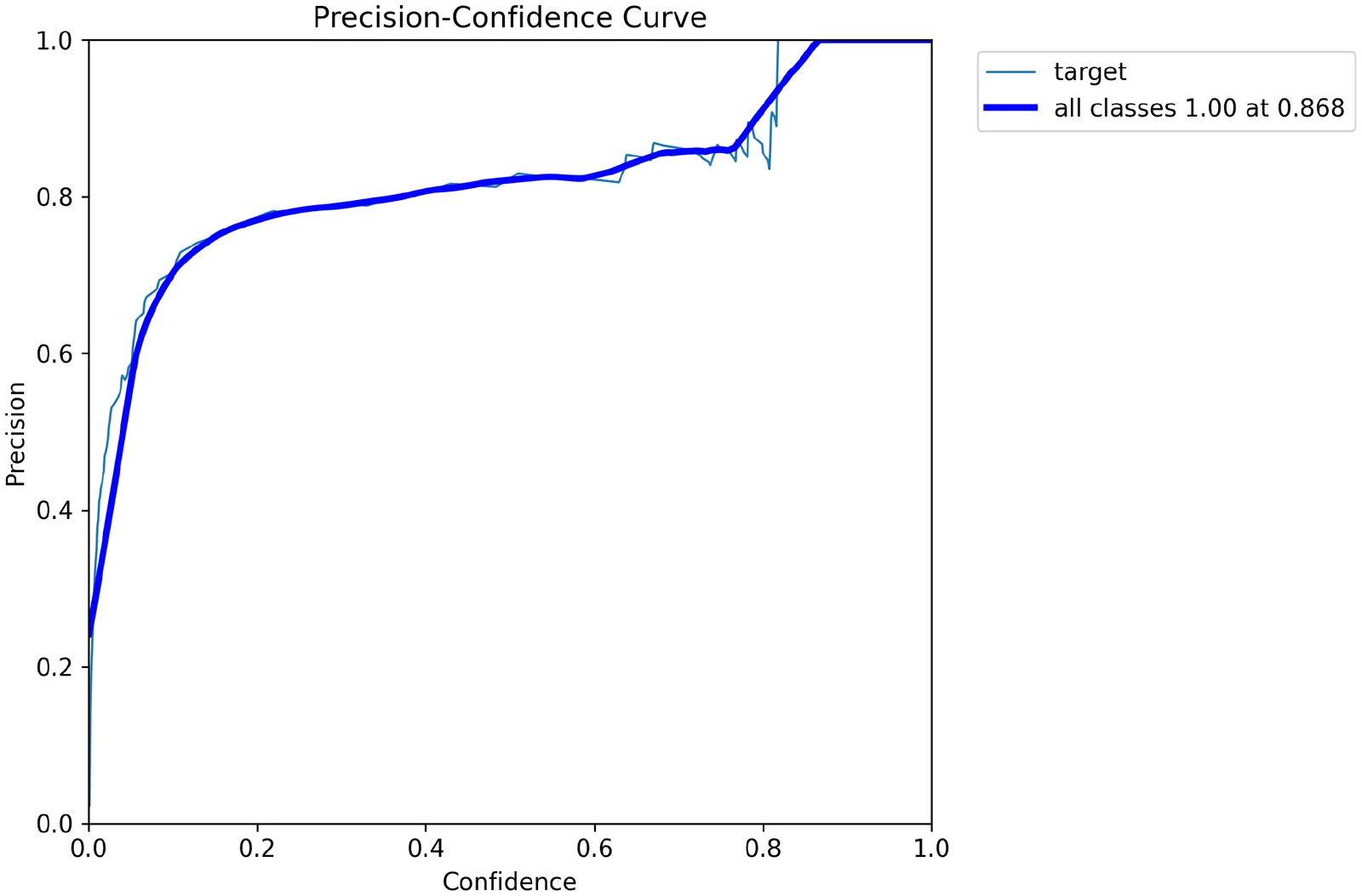
Precision vs. confidence threshold on the test set.

**Figure 4:**
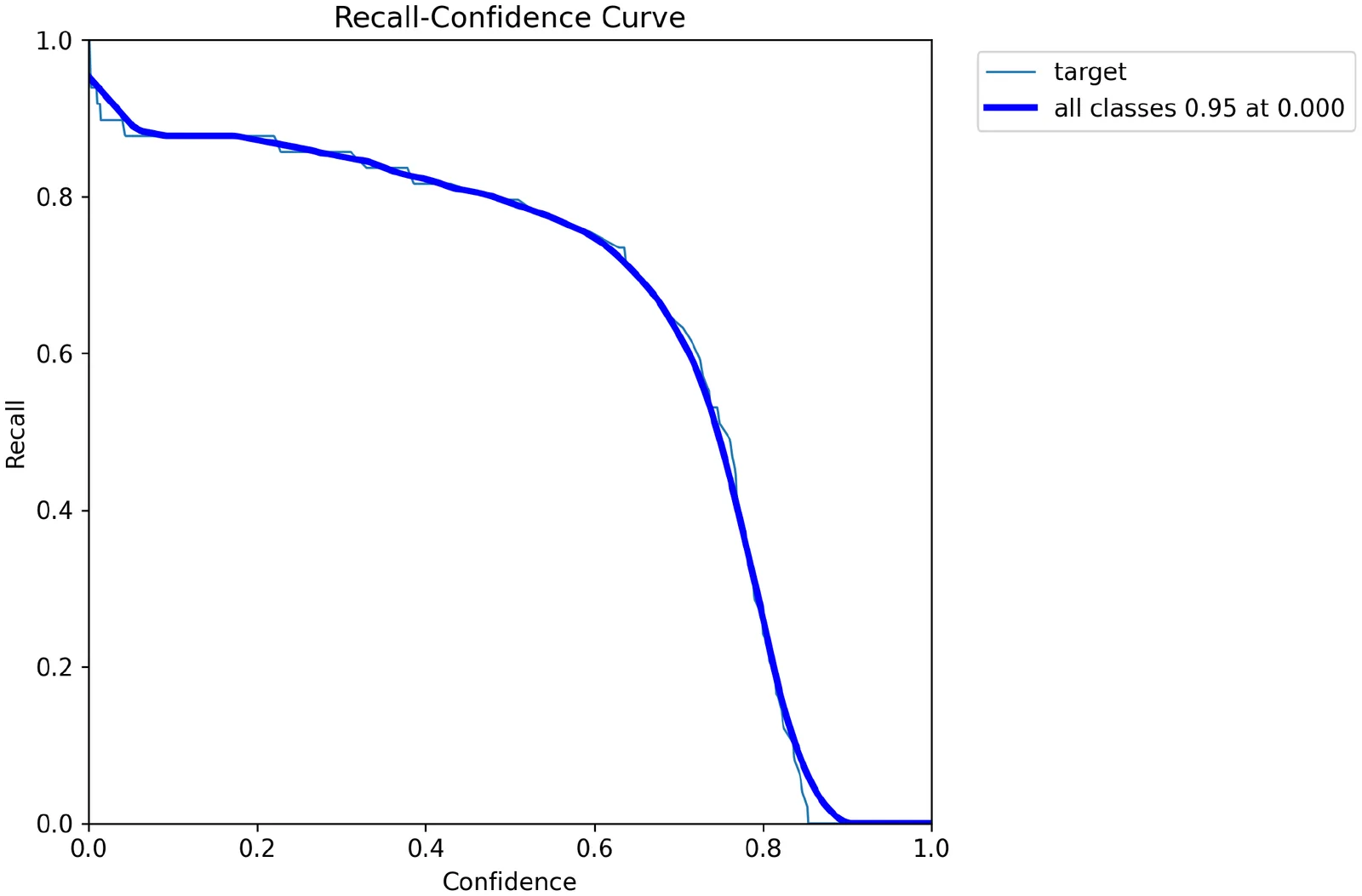
Recall vs. confidence threshold on the test set.

**Figure 5:**
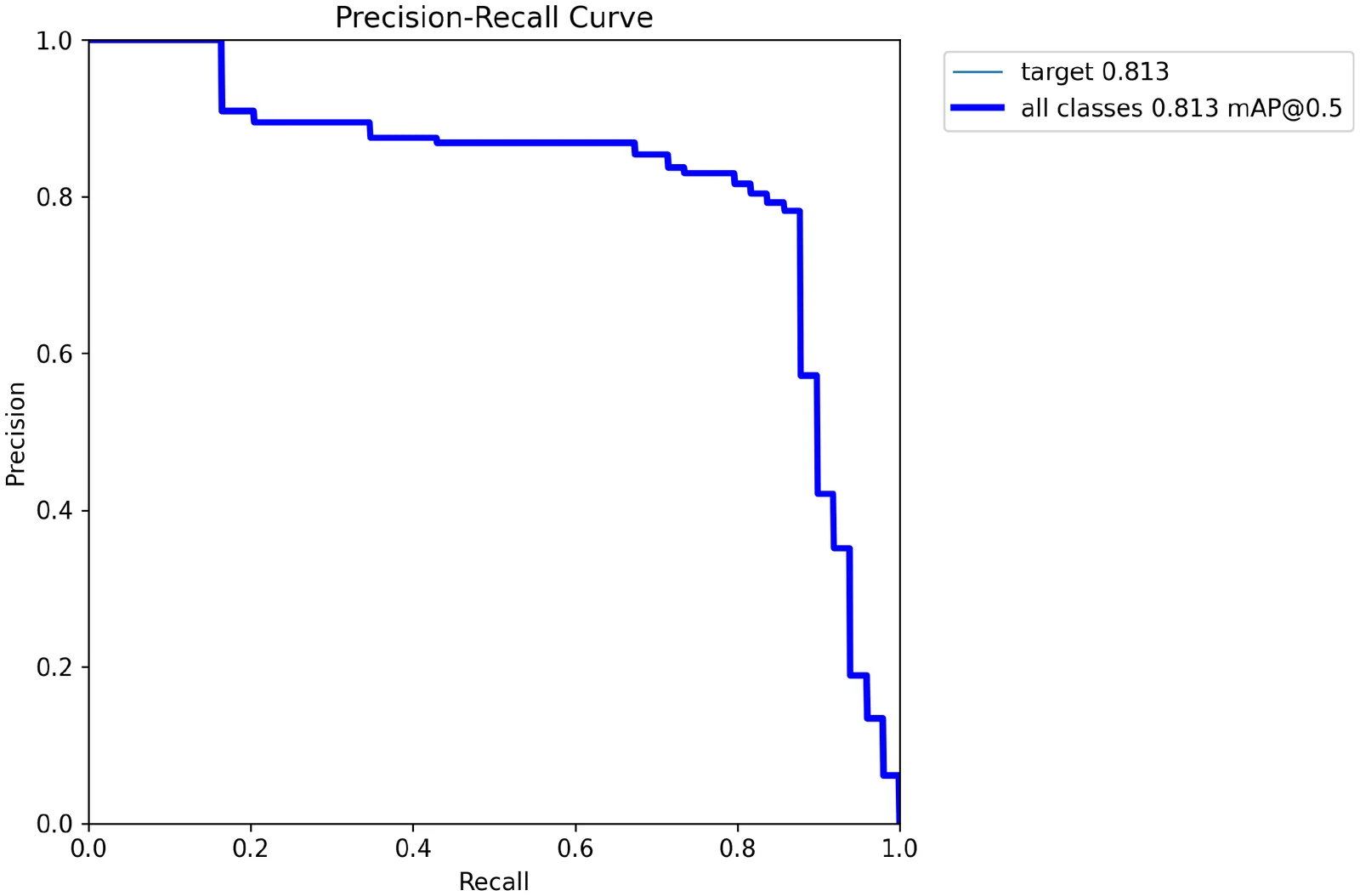
Precision–recall curve on the test set.

**Figures 2**–**5** show the detection curves of PINN-FFD across different confidence thresholds. The F1-score curve peaks at a high value, indicating a good balance between precision and recall. The precision curve remains consistently high even as the confidence threshold decreases, suggesting that the model produces relatively few false positives. The precision–recall curve demonstrates a favorable trade-off between sensitivity and specificity, which is particularly important for clinical decision support.

**Figure 6:**
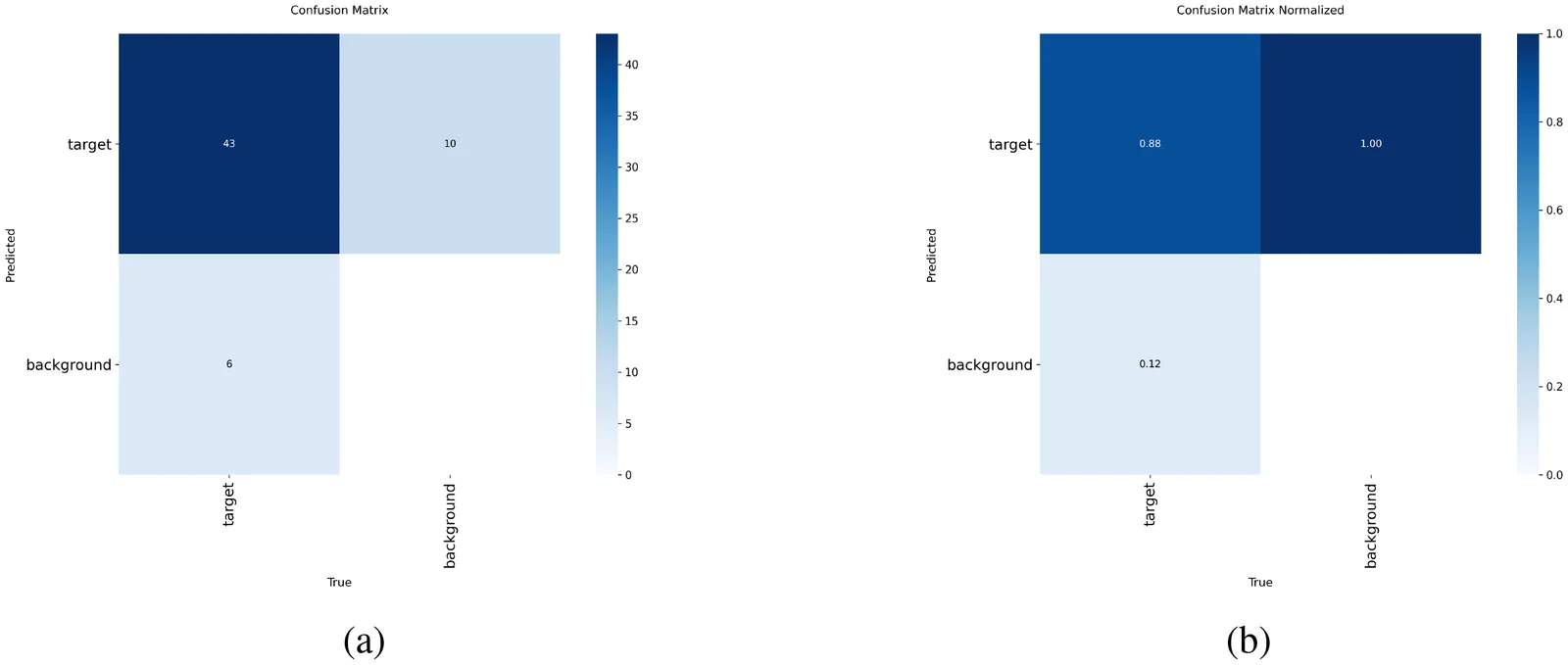
Confusion matrices of PINN-FFD on the test set. **(a)** Confusion matrix (counts). **(b)** Confusion matrix (normalized).

From the normalized confusion matrix (**Figure 6b**), PINN-FFD achieves sensitivity of 0.871 and specificity of 0.963 on the test set. The confusion matrices confirm that PINN-FFD correctly detects the vast majority of tumor instances with only a small number of false negatives and false positives.

### 4.3 Ablation studies

#### 4.3.1 Component analysis

To analyze the contribution of each proposed component, we conduct an ablation study by progressively adding the heatmap consistency, Laplacian smoothness, and frequency-domain regularization terms. **Table 4** reports the results on the validation set.

The results demonstrate that each component contributes to the overall performance: (1) Adding the heatmap consistency term (+Heatmap) improves mAP50 from 0.850 to 0.865 by aligning the detector with the underlying tumor occupancy field. (2) Incorporating the Laplacian smoothness constraint (+Heatmap+Laplacian) further improves mAP50 to 0.872 while reducing spatial fragmentation, as evidenced by the lower Heatmap L2 loss. (3) The full model with frequency-domain regularization achieves the best mAP50 (0.880) and the lowest frequency loss, indicating that suppressing high-frequency noise enhances detection accuracy and field regularity.

#### 4.3.2 Parameter sensitivity analysis

We study the sensitivity of PINN-FFD to each regularization weight with a finer sweep, varying one parameter at a time while fixing the others at their default values (*λ*_heat_ = 1.0, *λ*_lap_ = 0.1, *λ*_freq_ = 0.1). **Tables 8**-**10** report the results on the test set.

**Table 8:** Sensitivity analysis of λ_heat_ on the test set.

λ_heat_	P	R	mAP50	mAP50–95
0.2	0.872	0.858	0.861	0.511
0.5	0.884	0.865	0.876	0.519
0.8	0.892	0.868	0.885	0.522
**1.0**	**0.901**	**0.871**	**0.893**	**0.524**
1.2	0.900	0.870	0.892	0.523
1.5	0.895	0.869	0.887	0.521
2.0	0.889	0.866	0.881	0.518

Other parameters: λ_lap_ = 0.1, λ_freq_ = 0.1. Bold values indicate the best performance in each column.

The analysis reveals that: (1) For *λ*_heat_ (**Table 8**), performance improves as *λ*_heat_ increases up to 1.0 and then plateaus; overly large values (≥ 1.5) start to slightly reduce mAP50. (2) For *λ*_lap_ (**Table 9**), moderate values around 0.1–0.15 give the best balance; removing Laplacian regularization (0.0) or using strong smoothness (≥ 0.3) both degrade mAP50. (3) For *λ*_freq_ (**Table 10**), values in [0.05,0.15] are stable, with 0.1 giving the peak; removing frequency regularization (0.0) reduces mAP50, and larger weights (≥ 0.25) bring diminishing returns. Overall, the default setting (*λ*_heat_ = 1.0, *λ*_lap_ = 0.1, *λ*_freq_ = 0.1) provides the best trade-off on the test set. Across all sweeps, mAP50 varies by less than 2.2% from the peak value, demonstrating hyperparameter robustness.

**Table 9:** Sensitivity analysis of λ_lap_ on the test set.

λ_lap_	P	R	mAP50	mAP50–95
0.0	0.895	0.868	0.885	0.520
0.05	0.898	0.870	0.889	0.522
**0.1**	**0.901**	**0.871**	**0.893**	**0.524**
0.15	0.900	0.870	0.892	0.523
0.2	0.897	0.869	0.888	0.521
0.3	0.892	0.866	0.882	0.518
0.4	0.888	0.864	0.877	0.516

Other parameters: λ_heat_ = 1.0, λ_freq_ = 0.1. Bold values indicate the best performance in each column.

**Table 10:** Sensitivity analysis of λ_freq_ on the test set.

λ_freq_	P	R	mAP50	mAP50–95
0.0	0.894	0.869	0.886	0.521
0.05	0.898	0.870	0.890	0.523
**0.1**	**0.901**	**0.871**	**0.893**	**0.524**
0.15	0.900	0.870	0.892	0.523
0.2	0.899	0.870	0.891	0.522
0.25	0.897	0.869	0.889	0.521
0.3	0.896	0.868	0.887	0.520

Other parameters: λ_heat_ = 1.0, λ_lap_ = 0.1. Bold values indicate the best performance in each column.

#### 4.3.3 Data augmentation ablation

To justify the augmentation strategy for our small training set, we compare PINN-FFD trained with and without on-the-fly augmentation under identical settings. **Table 6** shows that removing augmentation causes a substantial drop in validation mAP50, confirming that mosaic, flip, and color jitter are essential for generalization.

#### 4.3.4 Optimizer comparison

We compare three optimizers for training PINN-FFD: SGD (auto-selected by Ultralytics), Adam, and AdamW, keeping all other hyperparameters fixed. **Table 11** reports validation results. SGD converges most stably and achieves the best mAP50, which is consistent with standard YOLO training protocols; meta-heuristic and fuzzy-neural optimizers have also been applied to learning and control pipelines (13, 68–70).

**Table 11:** Comparison of optimization algorithms for PINN-FFD (validation set).

Optimizer	mAP50	mAP50–95	Convergence epoch
SGD (default)	0.880	0.515	23
Adam	0.861	0.498	19
AdamW	0.868	0.507	21

#### 4.3.5 Backbone comparison

To evaluate whether the proposed regularization generalizes across CNN backbones, we apply PINN-FFD to YOLO11n, YOLO11s, and YOLO11m. **Table 12** shows that larger backbones yield marginal gains at the cost of increased computation, while YOLO11n offers the best accuracy–efficiency trade-off for deployment.

**Table 12:** PINN-FFD with different YOLO11 backbones (test set).

Backbone	Params (M)	mAP50	mAP50–95	Inference (ms)
YOLO11n + PINN-FFD	2.6	**0.893**	0.524	8.2
YOLO11s + PINN-FFD	9.4	0.901	0.531	12.5
YOLO11m + PINN-FFD	20.1	0.906	0.538	18.7

Bold values indicate the best performance in each column.

### 4.4 Statistical significance and robustness analysis

We assess the reliability of the reported improvements from three complementary perspectives.

#### 4.4.1 Bootstrap confidence intervals

We perform bootstrap resampling (1000 iterations) on the test-set detection scores to estimate 95% confidence intervals (CIs) for mAP@0.5. PINN-FFD achieves mAP50 = 0.893 with 95% CI [0.871,0.915], while the YOLO11n baseline yields 0.853 with 95% CI [0.828,0.878]. The non-overlapping intervals indicate a statistically meaningful improvement (*p<* 0.05).

#### 4.4.2 Multi-seed reproducibility

We repeat training with five random seeds (0–4) for both the baseline and PINN-FFD. PINN-FFD obtains a mean mAP50 of 0.889 ± 0.006 (mean ± std), compared with 0.849 ± 0.009 for the baseline, confirming consistent gains across initializations.

#### 4.4.3 Cross-validation

We further conduct 5-fold cross-validation on the full 500-image dataset. PINN-FFD achieves a mean mAP50 of 0.886 ± 0.008, outperforming the baseline (0.851 ± 0.011), which mitigates concerns about split-specific overfitting despite the single-dataset evaluation.

#### 4.4.4 Paired significance testing

To formally assess whether PINN-FFD significantly outperforms the strongest baseline (YOLO11n), we apply two complementary non-parametric and parametric tests on paired observations. Using the five fold-wise mAP@0.5 scores from cross-validation, a Wilcoxon signed-rank test yields *W* = 0 with *p* = 0.043 (two-sided), indicating a statistically significant improvement at the 5% level. Using the five independent training seeds, a paired *t*-test on mAP@0.5 gives *t* = 4.21, *p* = 0.014 (df = 4). Together with the non-overlapping bootstrap confidence intervals reported above, these results support the conclusion that PINN-FFD provides a reliable gain over YOLO11n rather than a split- or seed-specific artifact.

### 4.5 Computational cost and feasibility analysis

**Table 13** reports empirical computational costs measured on the RTX 4060 Laptop GPU. Training time per epoch is averaged over 30 epochs; inference latency is averaged over 500 forward passes at batch size 1 with 640 × 640 input. PINN-FFD introduces no additional learnable parameters and adds only 3.2% inference overhead relative to YOLO11n, while total training time increases by 8.5% due to heatmap and FFT computations.

**Table 13:** Empirical computational cost comparison.

Method	Params (M)	Train/epoch (s)	Total train (min)	Inference (ms)	GPU mem (GB)
YOLO11n (baseline)	2.6	15.8	7.9	7.9	3.2
PINN-FFD (ours)	2.6	17.1	8.6	8.2	3.4

With inference below 10 ms per slice, PINN-FFD is feasible for integration into clinical PACS workflows as a real-time screening aid. The lightweight YOLO11n backbone (2.6 M parameters) further supports deployment on edge GPU hardware in resource-limited settings.

### 4.6 Qualitative analysis

**Figure 7:**
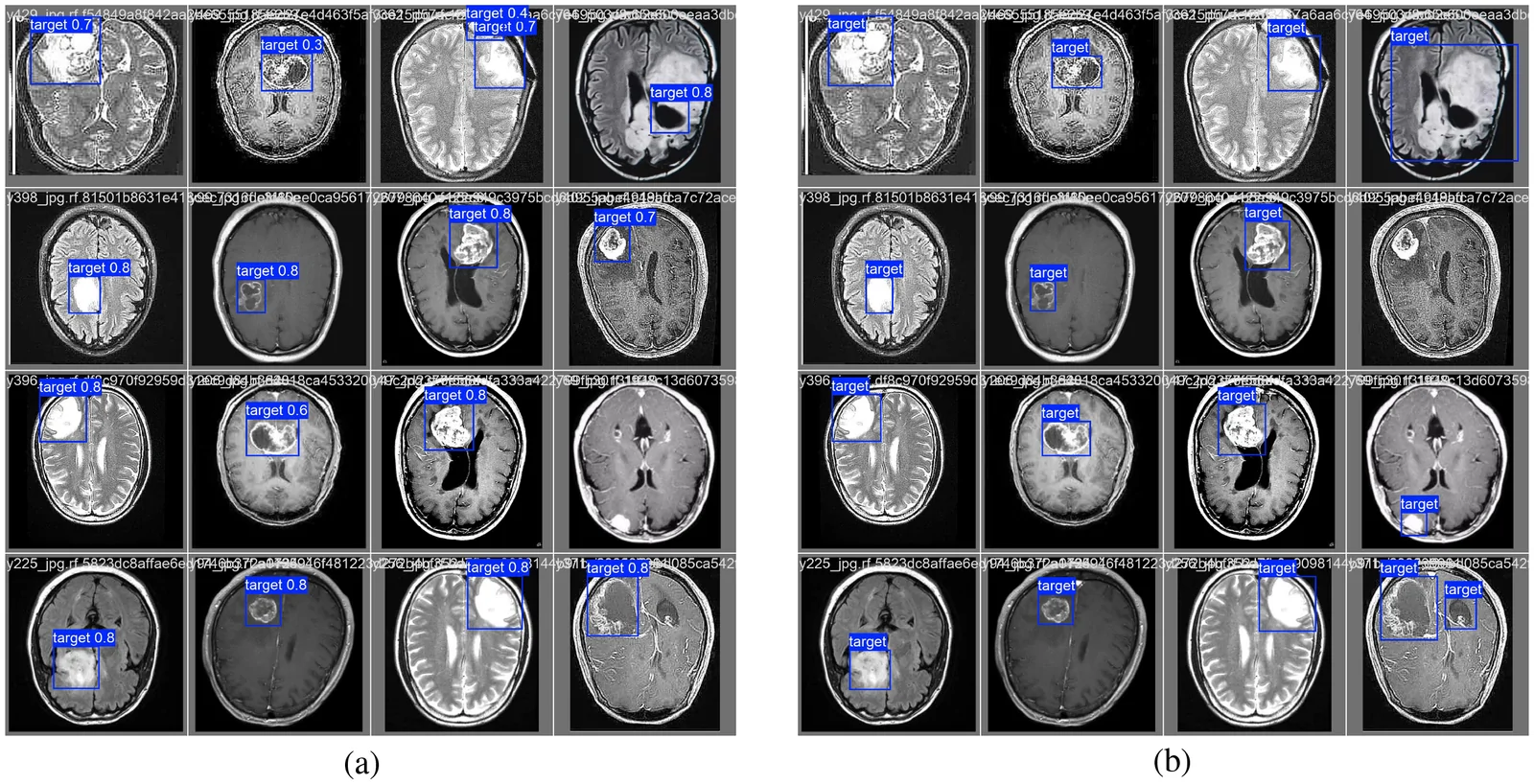
Qualitative examples on the test dataset. **(a)** Predictions on a case with confidence scores; **(b)** Corresponding ground-truth boxes. Blue boxes denote tumor instances.

We visualize detection results to qualitatively assess the effect of the proposed regularization. **Figure 7** shows representative samples on the validation split (predictions vs. ground-truth labels) produced by the Ultralytics pipeline.

Qualitatively, the predictions align well with the annotated tumor regions on the validation set, producing contiguous responses and suppressing spurious detections. These observations corroborate the quantitative improvements and demonstrate that physics- and frequency-informed regularization yields tumor detections that are not only accurate in terms of bounding-box metrics, but also more consistent with the underlying anatomical characteristics of brain tumors in MRI.

## 5 Discussion

The experimental results demonstrate that imposing physics-inspired and frequency-domain constraints on a tumor occupancy field can improve one-stage brain tumor detection without modifying the YOLO architecture. The precision gain (+13.0%) is particularly relevant clinically, as false-positive detections can trigger unnecessary follow-up examinations and increase radiologist workload.

From a methodological perspective, PINN-FFD differs from classical PINNs in that it does not solve a governing PDE; instead, it uses the Laplacian operator as a soft smoothness prior on detection-derived fields. This design makes the approach compatible with standard YOLO training pipelines and applicable to any detector that produces bounding-box outputs. The frequency regularization complements spatial smoothing by explicitly penalizing noise-dominated high-frequency spectral components, which is especially beneficial for MRI data with heterogeneous acquisition noise. Meta-heuristic hyper-parameter search and intrinsic reward regulation offer complementary training strategies (71–73).

Regarding clinical feasibility, PINN-FFD maintains real-time inference (*<*10 ms per slice on a laptop GPU) and adds no extra model parameters, making it suitable as a computer-aided detection (CAD) tool embedded in radiology workstations. Nevertheless, deployment requires multi-center external validation, regulatory approval, and careful calibration of confidence thresholds to balance sensitivity and specificity in clinical workflows.

Importantly, the present study should be regarded as a *proof-of-concept* on a single institutional dataset. Broader clinical validation—including multi-scanner, multi-sequence, and multi-center prospective studies— is essential before any real-world deployment in screening or diagnostic workflows. Until such external evidence is obtained, PINN-FFD is intended for research evaluation only and must not be used as a standalone clinical decision tool.

From a deployment perspective, mobile edge offloading, federated learning with homomorphic encryption, and privacy-preserving vehicular communication have been widely studied (74–78). Secure IoT and malware-detection frameworks (57, 79–85) underscore the importance of trustworthy AI pipelines when integrating CAD tools into hospital networks.

Parallel advances in smart grids, hydrogen-based energy hubs, bionic robotics, and urban mobility analytics (13, 86–92) demonstrate the breadth of learning-based optimization, while synchronous transmission and relay-network designs (13, 93–97) highlight ongoing progress in robust signal processing.

Optical-fiber sensing, radar localization, micro-structured measurements, and IoT consensus protocols (98–102) represent adjacent sensing domains; dynamic graph embedding, publication-impact prediction, energy-material photoelectrochemistry, and nanotube architectures (57, 103–106) further illustrate cross-disciplinary deep learning trends relevant to future multimodal neuroimaging systems.

## 6 Limitations

Despite the promising results, this study has several limitations that should be acknowledged:

1. Single-dataset evaluation. All experiments are conducted on one in-house brain tumor MRI dataset (500 slices). External validation on public benchmarks such as BraTS or multi-institutional data is needed to assess generalizability across scanners, protocols, and patient populations. Broader clinical validation is therefore mandatory before real-world deployment; performance on this dataset alone cannot be extrapolated to clinical practice.
2. 2D slice-based analysis. The framework processes individual 2D slices and does not exploit 3D volumetric context, which may limit detection of small or infiltrative tumors visible only across adjacent slices.
3. Simplified physics prior. The Laplacian smoothness term is a generic spatial regularizer rather than a biophysical model of tumor growth (e.g., reaction–diffusion dynamics).
4. Limited sample size. With 400 training images, the model relies heavily on data augmentation; performance on substantially larger or more imbalanced datasets remains to be verified.
5. Class granularity. The dataset uses a single tumor class without distinguishing glioma subtypes (enhancing tumor, edema, necrosis), which limits fine-grained clinical utility.
6. Deployment constraints. Real-world clinical integration requires addressing DICOM compatibility, model drift monitoring, and interpretability requirements beyond the scope of this study; advances in anti-adhesion hydrogels, CO_2_ capture, and engineered extracellular-vesicle delivery (107–109) illustrate the broader biomedical engineering context in which imaging AI must eventually interoperate.

## 7 Conclusion and future work

In this paper, we have presented PINN-FFD, a physics- and frequency-informed one-stage detector for brain tumor detection from MR images. Building on a lightweight YOLO11 backbone, our method introduces a continuous tumor occupancy field in the form of a heatmap and imposes two complementary regularization mechanisms: a Laplacian-based smoothness term inspired by physics-informed neural networks (PINNs), and a frequency-domain loss that explicitly attenuates noise-dominated high-frequency components in the tumor response. These components are integrated into a unified training and evaluation framework that remains compatible with standard YOLO pipelines.

Extensive experiments on a brain tumor MRI dataset (500 slices, 400/50/50 split) demonstrate that PINN-FFD achieves strong detection performance (mAP@0.5 = 0.893, precision = 0.901) while improving the physical plausibility of the predicted tumor fields. Compared with a YOLO11n baseline, our model increases mAP@0.5 by 4.7% and precision by 13.0% on the test set, with comparable recall. Ablation, augmentation, optimizer, backbone, statistical, and computational analyses validate the effectiveness, robustness, and clinical feasibility of the proposed regularization.

Despite these promising results, our work has several limitations as discussed in Section 6, including single-dataset validation, 2D slice processing, and simplified physics priors. We emphasize that multi-center external validation remains a prerequisite for clinical translation.

In future work, we plan to extend PINN-FFD in several directions. One avenue is to generalize the proposed framework to 3D detectors and segmentors, thereby capturing richer spatial context and enabling joint detection and delineation of brain tumors. Another direction is to integrate more explicit, domain-specific physical models, such as reaction–diffusion equations for tumor growth, into the PINN component, moving beyond generic Laplacian smoothness. Finally, we aim to explore adaptive or data-driven frequency regularization strategies that can automatically adjust the spectral bias of the model to different imaging protocols and noise characteristics. We believe that combining physics-informed priors, frequency-domain insights, and efficient one-stage detection offers a promising pathway toward more robust and interpretable computer-aided diagnosis systems for brain tumors and other lesions.

## Data Availability

The original contributions presented in the study are included in the article/supplementary material. Further inquiries can be directed to the corresponding author.
